# Use of Respondent Driven Sampling (RDS) Generates a Very Diverse Sample of Men Who Have Sex with Men (MSM) in Buenos Aires, Argentina

**DOI:** 10.1371/journal.pone.0027447

**Published:** 2011-11-10

**Authors:** Alex Carballo-Diéguez, Ivan Balan, Rubén Marone, María A. Pando, Curtis Dolezal, Victoria Barreda, Cheng-Shiun Leu, María Mercedes Ávila

**Affiliations:** 1 HIV Center for Clinical and Behavioral Studies, New York State Psychiatric Institute and Columbia University, New York, New York, United States of America; 2 Nexo Asociación Civil, Buenos Aires, Argentina; 3 Centro Nacional de Referencia para el SIDA, Departamento de Microbiología, Parasitología e Inmunología, Facultad de Medicina, Universidad de Buenos Aires, Buenos Aires, Argentina; McGill University AIDS Centre, Canada

## Abstract

**Background:**

Prior research focusing on men who have sex with men (MSM) conducted in Buenos Aires, Argentina, used convenience samples that included mainly gay identified men. To increase MSM sample representativeness, we used Respondent Driven Sampling (RDS) for the first time in Argentina. Using RDS, under certain specified conditions, the observed estimates for the percentage of the population with a specific trait are asymptotically unbiased. We describe, the diversity of the recruited sample, from the point of view of sexual orientation, and contrast the different subgroups in terms of their HIV sexual risk behavior.

**Methodology:**

500 MSM were recruited using RDS. Behavioral data were collected through face-to-face interviews and Web-based CASI.

**Conclusion:**

In contrast with prior studies, RDS generated a very diverse sample of MSM from a sexual identity perspective. Only 24.5% of participants identified as gay; 36.2% identified as bisexual, 21.9% as heterosexual, and 17.4% were grouped as “other.” Gay and non-gay identified MSM differed significantly in their sexual behavior, the former having higher numbers of partners, more frequent sexual contacts and less frequency of condom use. One third of the men (gay, 3%; bisexual, 34%, heterosexual, 51%; other, 49%) reported having had sex with men, women *and* transvestites in the two months prior to the interview. This population requires further study and, potentially, HIV prevention strategies tailored to such diversity of partnerships. Our results highlight the potential effectiveness of using RDS to reach non-gay identified MSM. They also present lessons learned in the implementation of RDS to recruit MSM concerning both the importance and limitations of formative work, the need to tailor incentives to circumstances of the less affluent potential participants, the need to prevent masking, and the challenge of assessing network size.

## Introduction

To recruit a “hidden population,” one for which no sampling frame exists and public acknowledgement of membership is potentially threatening or stigmatized, is always a challenge. This is frequently the case when attempts are made to recruit men who have sex with men (MSM), especially if the researchers are trying to obtain representative samples. Time-space sampling [1] and random digit dialing [2] have been used to sample MSM, but these methods are very costly and carry their own biases. Time-space sampling has external validity for populations who attend the venues included in the sampling frame, but not for others [3] –e.g., men who socialize mainly on the Internet are not reached by geographical sampling. Random digit dialing generates biased samples when socioeconomic conditions result in many dwellings with no phones or where mobile phones or voice over Internet replace landlines.

An increasingly popular sampling alternative is Respondent Driven Sampling (RDS) [4], [5], a method for data collection and statistical inference. A small number of participants (referred to as “seeds”) are recruited by the investigators both to participate in the study and to invite a limited number of people (usually no more than three) from their network to enroll as well. Participants give coupons to those they invite. Each subsequent participant can also recruit people from his network until the target number is reached. All participants are offered a dual incentive (for participation and for each person they recruit) thus producing chains of referrals that move away from the initial seeds. All participants are asked to report the number of people in their network who belong to the target population; this number is used to weight the provided data. A review [6] of more than 120 RDS studies conducted worldwide found that RDS is an effective technique, when designed and implemented appropriately, to sample most-at-risk populations for human immunodeficiency virus (HIV) biological and behavioral surveys. Although a recent publication raises questions about the suitability of RDS for key aspects of public health surveillance [7], at the time of our study several prior ones had demonstrated that, under certain specified conditions, the observed estimates for the percentage of the population with a specific trait are asymptotically unbiased [8] (i.e., essentially correct in large samples with high probability).

Prior research focusing on MSM conducted in Buenos Aires, Argentina, used community outreach strategies and non-probability sampling from bars and other social venues. This resulted in samples of mainly gay identified men. This was the case in a cross-sectional study conducted in 2001 [9] and a cohort study enrolled in 2003 [10]. Studies of MSM conducted in other parts of the world employing RDS resulted in samples of MSM that included important proportions of bisexual men [11]–[15]. To increase the representativeness of the MSM participating in our study, we used RDS for the first time in Argentina to recruit MSM. The purpose of this manuscript is to describe, from the point of view of sexual orientation, the diversity of the sample of MSM recruited through RDS and to contrast the different subgroups in terms of their HIV sexual risk behavior.

## Methods

### Ethics Statement

This research study received approval from the Institutional Review Boards of the New York State Psychiatric Institute, New York, NY, USA, and the Comité Independiente de Ética en Investigación de la Facultad de Medicina, Universidad de Buenos Aires, Buenos Aires, Argentina. Written consent was obtained from all participants prior to enrollment in the study. All data from this study will be de-identified prior to making it available to other researchers once data analysis is concluded.

### Stages of the study

The field name of this study (chosen by the Buenos Aires-based researchers) was “Links,” a reference to the chains of MSM expected to participate. It consisted of a formative stage (as recommended by RDS specialists) [16], followed by a survey stage.

The objectives of the formative stage were to gather and elicit sufficient information on MSM in Buenos Aires to determine the appropriateness of RDS for the recruitment to take place in the second stage of the study, to determine appropriateness of incentives, and to tailor or develop research instruments for the survey. In this stage, 1) we conducted a review of the available scientific and lay literature on MSM in Buenos Aires; 2) we interviewed individually ten local system representatives (public health functionaries, HIV health care providers, gay non-governmental organization –NGO– representatives, and sex researchers) and 16 key informants (bartenders, party organizers, sex workers); 3) trained ethnographers supervised by an anthropologist undertook observations of public and private places frequented by MSM (bars, discos, hotels, porno theaters, dark rooms, parks, public toilets, private parties, and cruising areas); 4) we held eight focus groups (with a total of 73 participants) stratified by HIV status (positive/negative), age (above/below 35 years), and education (high school graduate or higher/below high school); and finally 5) we interviewed individually 18 MSM who did not identify as gay. (Some of these interviews took place in the second stage of the study). Partial results of this formative stage appear elsewhere [17].

The survey stage of the study was conducted using RDS. Eligibility criteria included to identify as a man, be 18 years or older, have had sex with another man or a male-to-female (MTF) transvestite (transvestite rather than transgender is the term used in Buenos Aires; the category includes both pre- and post-operative individuals) in the prior six months, have had sex with a man (or men) or MTF transvestite(s) at least 10 times in his lifetime, reside in the Buenos Aires or its suburban areas, have a coupon received from a prior participant (not applicable to seeds), and agree to provide a blood sample for HIV and STI testing. MTF transvestites were not included in this survey.

### Procedure

Seeds were recruited based on their purported large networks of MSM acquaintances, likelihood of referring three of them to the study, diversity of backgrounds, self-reported serostatus (either infected or not), sexual identity, and availability. They were selected by NGO personnel who knew them from participation in organization activities or had met them during the formative stage of the study. When some seeds failed to refer participants, additional seeds were recruited. A total of 16 seeds were recruited. They underwent all study procedures and were then given three coupons (valid for 60 days) to pass on to members of their networks. In several cases, participants returned to the study site accompanying their three recruits. In total, 500 MSM (our target sample by study design) were recruited from November 2007 through July 2009.

All participants were seen at the offices of Nexo Asociación Civil, our NGO partner. The offices are in one centrally located building easily accessible by public transportation. Individuals who received coupons could call for an appointment or present themselves at the research offices where a research assistant (always the same person to recognize individuals attempting to re-enroll) screened men for eligibility. When someone did not qualify, the coupon was retained –it could be given back to the referring participant so that he could give it to someone else– but the person was nevertheless offered HIV testing and counseling. Qualifying men underwent consent and proceeded to respond to a password protected, Web-based computer assisted self-interview (CASI) that inquired, among other topics, about demographic information and recent sexual behavior. Participants unable to use the computer received help from a research assistant who read the questions verbatim and entered the answers. As part of the study, blood was drawn for HIV, hepatitis C virus (HCV), hepatitis B virus (HBV) and T. pallidum testing. Blood tests were a requirement for study participation to which participants agreed in the consent process. Pre-HIV counseling was provided to all participants and post-HIV counseling to those who returned to get their results. Participants could also choose to provide anal cells collected with a cytobrush for human papillomavirus (HPV) and Chlamydia diagnoses (HIV and sexually transmitted infection (STI) data will be reported elsewhere). Finally, the research assistant administered the Social Network Assessment, handed the participants three coupons, encouraged each participant to “give the coupons to people like you,” and took contact information to inform participants when they could cash the compensation for participant referral. Altogether, the interviews lasted from two to three hours. At the end of the interview, each participant received the equivalent of 20 United States dollars (USD) (equivalent to the cost of five movie tickets) as compensation for his time. For each referred acquaintance who qualified for the study, regardless of whether he enrolled or not, the participant received an additional USD 5.

### Instruments

#### Demographics and Sexual Orientation

This questionnaire covered age, education, income, work status, residence, civil status, and health insurance. Separately, participants were asked if they considered themselves gay/homosexual, bisexual, transvestite, heterosexual, or other.

#### Social Network

The following questions, posed by a research assistant, were used to determine size of social network: 1) Approximately, how many men who have sex with other men and/or transvestites do you know personally? 2) Of those men, how many do you know by name, who they are, and how to contact them? 3) Of those men, how many also know you? 4) Of these men, with how many have you been in contact in the past six months? Contact may be face-to-face, by phone, or email. 5) Of those men, how many live in Buenos Aires? 6) Of those men, how many are 18 years or older? 7) Of these men, how many could be willing to participate in this study? 8) Who gave you the coupon to participate in this study (study personnel, friend, partner, family member, workmate, someone unknown, other)? We used question 7 to measure network size for the purpose of RDS weighting.

#### Sexual behavior

In three separate questions, participants were asked if they had had sexual intercourse with men, women or MTF transvestites in the prior year. More detailed questions followed focusing on behavior in the prior two months with each partner type. Questions focused on number of partners and occurrence of oral, vaginal or anal sex; insertive or receptive intercourse; condom use; and, in cases of condomless intercourse, assumed HIV status of partners.

### Statistical analysis

Unlike a conventional probability sampling design in which each unit has a known and constant probability of selection, in RDS each person sampled does not have the same probability of being included in the sample: rather, persons with larger personal networks have a greater likelihood of being sampled than those with smaller personal networks. (For a full explanation, see Salganik and Heckathorn [8]). RDS takes this into consideration by weighting data based on reported network size. In our study, weights were calculated as the inverse of the participant's personal network size (PNS). This value was then multiplied by the sample size (N) divided by the sum of weights (∑w). The weighting formula is then:
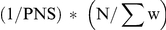



This weighting estimator is based on the RDS II estimator [18]. This produces results that reflect the original sample size of 500. Except for Table 1, which shows actual (i.e., unweighted) values in order to demonstrate actual recruitment numbers, all subsequent tables and statistical analyses are based on weighted data. Data were weighted prior to analyses. All statistical analyses were conducted using SPSS [19]. The four sexual identity groups were compared in terms of demographics and the prevalence of sexual risk behavior using chi-square tests (for dichotomous and categorical variables) and ANOVAs (for continuous variables). The Holm step-down procedure was used to adjust for multiple comparisons on tests for sexual risk behaviors. For some analyses, if 4-group comparisons were statistically significant, 2-group comparisons followed using chi-square and t-tests to compare gay identified participants to all non-gay identified men (not presented in the tables). In addition, the demographic characteristics of our sample were compared (separately) to two previously reported samples. These categorical variables were compared using Fisher's Exact tests.

**Table 1:** Yield of 16 seeds enrolled in RDS (unweighted data).

No. Seeds	No. referrals	Total referrals
7	0	0
4	1	4
1	3	3
1	4	4
1	5	5
1	38	38
1	430	430
Total 16		484

## Results

### Recruitment

**Figure 1:**
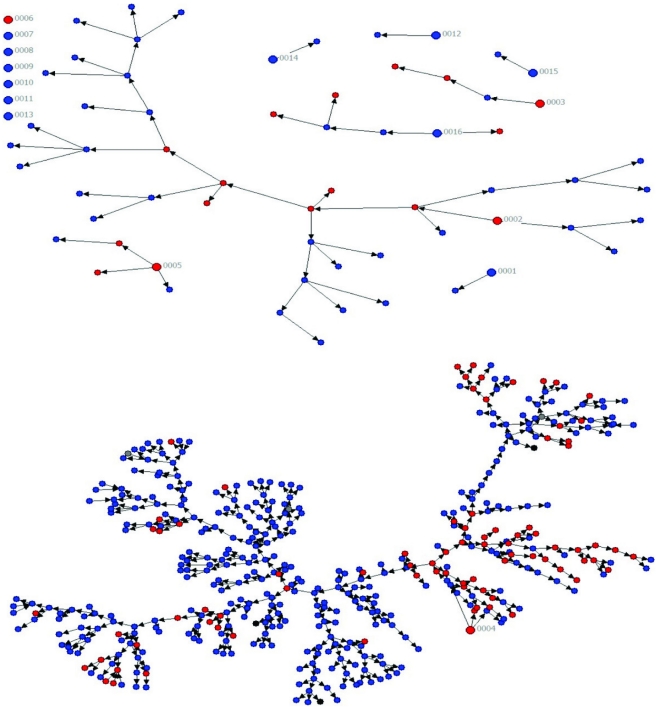
Referral networks generated by RDS (N = 500). Key: Large circle  =  seed; red  =  HIV infected; blue  =  HIV uninfected Note: Figure 1 was generated with unweighted values.

Of the 16 seeds, seven (all but one HIV infected by self report) did not recruit any participants. The yield of all seeds is presented in Table 1. It should be noted that one seed generated 430 referrals or 86.0% of the sample (see Fig. 1). This long chain reached 22 waves or iterations of referrals. Table 1 shows actual (i.e., unweighted) values to facilitate comparison with Fig. 1. All subsequent descriptive tables and statistical analyses are based on weighted data.

### Demographics and sexual orientation

Of the 500 MSM in the sample, only 24.5% identified as gay, whereas 36.2% identified as bisexual, 21.9% as heterosexual, and 17.4% as other. The latter tended to be younger and of lower educational level. They wrote in labels such as “hombre” (man), “macho,” and “activo” (active) that did not allow clear assignment to any of the other three categories.

Table 2 presents the demographic characteristics of the sample by sexual identity. Participants' mean age was 30.5 (SD 11.5); almost half of participants were in the 18- to 25-year-old bracket. Two thirds had not completed high school with lower levels of education being reported by the non-gay identified men. In terms of income, 29% had none, with non-gay identified men earning less than gay identified men. Almost one third of participants reported being unemployed, and a similar proportion only had temporary work (categories could overlap). Twenty-nine percent resided in the Ciudad Autónoma de Buenos Aires (Buenos Aires proper, the capital city) while the majority of participants came from the less affluent suburbs of the West Zone and South Zone. Most participants were single. Four out of 5 participants had no health insurance. Participants' average network size was 2.89 persons (range 1-50, SD = 2.01), with gay identified men having the largest network size and “other” the smallest. In 91.8% of cases participants were referred by friends or acquaintances. In summary, this was a sample of young, mostly non-gay identified MSM of low socioeconomic status (especially among the non-gay identified), high unemployment, living mainly in the less affluent areas surrounding Buenos Aires, mainly lacking health insurance, and socially connected to other MSM.

**Table 2:** Demographic characteristics of 500 MSM recruited through RDS (weighted values).

	Total (N = 500)	Gay (n = 123)	Bisexual (n = 181)	Hetero (n = 109)	Other (n = 87)	F/X^2^	df	p
	N (%)	n (%)	n (%)	n (%)	n (%)			
**Age** a								
18-25	232 (47)	37 (30)	83 (46)	58 (54)	54 (64)	5.82	3	.001
26-35	104 (21)	26 (21)	43 (24)	19 (18)	16 (19)			
36-45	104 (21)	43 (35)	37 (21)	16 (15)	8 (10)			
46-55	35 (7)	10 (8)	2 (7)	10 (9)	3 (4)			
56+	19 (4)	7 (6)	5 (3)	4 (4)	3 (4)			
**Education** a								
Primary school or less	154 (31)	20 (16)	52 (29)	42 (39)	40 (48)	31.23	3	<.001
Incomplete HS	171 (35)	19 (15)	83 (46)	34 (32)	35 (42)			
Completed HS	91 (18)	33 (27)	28 (16)	22 (21)	8 (10)			
Some tertiary studies	68 (14)	40 (33)	18 (10)	9 (8)	1 (1)			
University degree	11 (2)	11 (9)	0 (0)	0 (0)	0 (0)			
**Monthly income (in pesos)** a								
None	112 (29)	24(23)	46 (32)	23 (27)	19 (32)	3.91	3	.009
Less than $1000	169 (43)	36 (35)	59 (41)	48 (57)	26 (43)			
$1000 - $1999	83 (21)	29 (28)	31 (21)	10 (12)	13 (22)			
$2000 - $2999	19 (5)	9 (9)	6 (4)	3 (4)	1 (2)			
$3000+	10 (3)	5 (5%)	3 (2)	1 (1)	1 (2)			
**Work status** b								
Temporary work	158 (32)	23 (19)	61 (34)	41 (38)	33 (39)	13.68	3	.003
Unemployed	151 (30)	37 (30)	43 (24)	38 (35)	33 (39)	7.53	3	.057
Employed by employer	126 (25)	51 (42)	44 (24)	13 (12)	18 (21)	28.24	3	<.001
Self-employed	109 (22)	26 (21)	44 (24)	21 (19)	18 (21)	1.19	3	.755
Student	89 (18)	37 (31)	33 (18)	10 (9)	9 (11)	21.55	3	<.001
**Place of residence** c								
Ciudad Autónoma de Buenos Aires	142 (29)	46 (38)	54 (30)	26 (24)	16 (18)	20.23	6	.003
West Zone	269 (54)	51 (42)	99 (55)	70 (64)	49 (56)			
South Zone	77 (15)	19 (16)	23 (13)	13 (12)	22 (25)			
North Zone	11 (2)	6 (5)	5 (3)	0 (0)	0 (0)			
**Civil status** d								
Single	387 (78)	101 (83)	146 (80)	74 (68)	66 (81)	9.24	3	.026
Married	19 (4)	1 (1)	14 (8)	1 (1)	3 (4)			
Other	89 (18)	20 (16)	22 (12)	34 (31)	13 (16)			
**Health insurance** e								
None	389 (79)	71 (58)	146 (82)	100 (92)	72 (88)	47.14	3	<.001
“Obra social”	91 (19)	44 (36)	29 (16)	9 (8)	9 (11)			
Pre-paid	13 (3)	8 (7)	4 (2)	0 (0)	1 (1)			
**Network size [Mean (SD)]**	2.87 (1.99)	3.30 (2.92)	2.98 (1.57)	2.53 (1.63)	2.32 (0.99)	4.92	3	.002

a **Age, education, and monthly income were analyzed as continuous variables (prior to collapsing into categories, if applicable) by ANOVA.**

b **Categories are not mutually exclusive so may sum to more than 100%.**

c **“North Zone” was excluded from the Chi-square test, due to empty or low N cells.**

d **“Married”/“Other” were compared to “Single” in Chi-square test.**

e **“Obra social”/“Pre-paid” were compared to “None” in Chi-square test.**

### Comparison with prior studies

Table 3 shows the comparison of the demographic data from our sample with those from two previous studies of MSM in Buenos Aires: Segura et al. [10] and Pando et al. [20]. The table shows that our sample was smaller than the other two. The most striking difference is the diversity in sexual orientation: whereas the majority of participants identified as gay in the previous studies (Pando et al. do not report proportion but describe the sample as “gay”), in our study only 24.5% of participants identified as gay. Also, our sample had more participants with lower levels of education than in the two other studies and more participants with no health insurance than in Segura's sample (Pando et al. did not report this variable). Yet, our sample had a higher proportion of participants with income in pesos higher than USD 100 per month and fewer unemployed participants than those in Segura's sample but more than in Pando's (see Discussion).

**Table 3:** Comparison of Links sample with previous studies on demographic parameters.

	Links^1^	Segura et al. (2007)				Pando et al. (2003)			
	N = 500	N = 877	Fisher	df	p	N = 694	Fisher	df	p
	n (%)	n (%)				n (%)			
**Education**			410.8	2	<.001		251.4	2	<.001
Primary	154 (32)	128 (15)				33 (5)			
Secondary	262 (55)	154 (18)				333 (48)			
Superior	62 (13)	595 (68)				328 (47)			
**Unemployed**	151 (30)	287 (33)	177.5	1	<.001	101 (15)	42.7	1	<.001
**Income (dollars per month)**			7.1	1	.009				
Low (≤100)	280 (72)	529 (79)^2^							
High (> 100)	112 (29)	143 (21)^2^							
**No Health Insurance**	389 (79)	427 (48)	126.6	1	<.001				
**Sexual Identity**			507.4	3	<.001				
Gay	123 (25)	736 (84)							
Bisexual	181 (36)	89 (10)							
Heterosexual	109 (22)	34 (4)							
Other	87 (17)	16 (2)							

1 **Weighted values**

2 **Percentages are corrected from original table.**

**Segura et al. sample was recruited in 2003; Pando et al. sample was recruited in 2000-2001.**

### Sexual Behavior

By eligibility criteria all men had had sex with another man at least once in the prior six months. At the time of the assessment, 88% of participants reported having had sex with a man in the prior two months (see Table 4), this being more frequent among gay identified men. Large proportions of non-gay identified men reported sex with a woman in the past two months, this being a rare occurrence among gay identified participants. Non-gay identified men also had had sex with MTF transvestites in the prior two months, ranging from 48% for bisexuals to 68% for heterosexuals. Interestingly, one third of the men reported that in the prior two months they had had sex with men, women *and* MTF transvestites, this being reported by half of the heterosexually identified men. Compared to non-gay identified participants, a higher proportion of gay identified men had had unprotected anal sex with a man in the prior two months. In terms of number of partners, 88% of participants had had more than one sexual partner in the past two months, with a median of 5.8 (SD 13.13) unprotected anal or vaginal intercourse occasions (range 0-200).

**Table 4:** Sexual behavior over the past two months by sexual orientation.

	Total (N = 500)	Gay (n = 123)	Bisexual (n = 181)	Hetero (n = 109)	Other (n = 87)	X^2^	df	p-value
	N (%)	n (%)	n (%)	n (%)	n (%)			
**Oral/anal sex w/man**	439 (88)	119 (97)	162 (90)	84 (76)	74 (87)	23.4	3	<.001
**Oral/anal/vaginal sex w/woman**	313 (63)	5 (4)	139 (77)	97 (88)	72 (85)	244.4	3	<.001
**Oral/anal sex w/transvestite**	226 (45)	11 (9)	88 (48)	74 (68)	53 (62)	97.9	3	<.001
**Sex with man, woman, ** ***and*** ** transvestite**	163 (33)	4 (3)	62 (34)	55 (51)	42 (49)	75.0	3	<.001
**Unprotected anal sex w/man**	205 (41)	71 (58)	74 (41)	32 (29)	28 (33)	22.9	3	<.001
**Unprotected anal or vaginal sex w/woman**	207 (44)	4 (3)	88 (52)	67 (63)	48 (62)	109.4	3	<.001
**Unprotected anal sex w/transvestite**	80 (17)	5 (4)	36 (21)	26 (25)	13 (16)	20.8	3	<.001
**Any unprotected anal or vaginal sex**	335 (68)	71 (58)	128 (71)	78 (74)	58 (68)	8.0	3	.045

**Since each contrast involves 8 comparisons, p values less than 0.006 are significant.**

## Discussion

In this study, the use of RDS resulted in a sample in which three quarters of MSM did not identify as gay. This was important to allow us to study a sector of the MSM population that is insufficiently represented in most convenience samples. Non-gay identified men differed in demographic characteristics, and sexual risk behavior, from gay identified men.

Although income was lower and unemployment higher in a prior MSM study [10], this may have been an effect of the economic turmoil that affected Argentina around the time Segura's sample was recruited (2003). In such circumstances, educational level may be a more stable and reliable socioeconomic indicator than income and employment for an adult sample of men. The educational level of our sample was lower than that in Segura's and Pando's samples, which seems to indicate that ours comes from a lower socioeconomic stratum of society. Furthermore, compared to Segura's study, a significantly larger proportion of our participants lacked any kind of health insurance, a further indicator of low socioeconomic status. From these perspectives, RDS appears to have been effective in reaching a “hidden” or hard-to-reach population of MSM in Buenos Aires.

From a sexual behavior perspective, as expected, men who identified as gay had fewer recent female or transvestite partners than the men who did not identify as gay. Consistent with the literature on sexual behavior among men in Latin America and Latin American men in the U.S. [21]–[25], we also expected that a significant portion of the MSM in the study would have sex with women. What we did not expect was the large proportion of men (one third of the sample) who reported having had sex with men, women, *and* transvestites in the past two months. This finding merits further exploration. It may be hypothesized that among young MSM of lower socioeconomic status in Buenos Aires contextual circumstances favor sexual partnerships with a high degree of flexibility and less subject to more traditional heterosexual/homosexual dichotomies. Alternatively, this finding may be an artifact of the recruitment process if men wishing to receive incentives exaggerated the range of their sexual activity or claimed to be MSM when they were not in the hope of entering the study. This has been reported in other MSM studies that used RDS [26]. However, in-depth interviews conducted in the course of the study did not find conclusive evidence of deception attempts. Furthermore, the ethnographic observations of MSM cruising sites in Buenos Aires that were conducted during the first phase of this study revealed numerous venues frequented by men, some wearing wedding bands, in which they could meet other men or transvestites for sex.

Unprotected intercourse was reported by the majority of participants. A higher proportion of gay identified men reported no or inconsistent condom use for anal intercourse than non-gay indentified men. The reverse was the case when both unprotected vaginal and anal intercourse were considered together: more non-gay indentified men had unprotected intercourse. Yet, these values cannot necessarily be considered equivalent to sexual risk behavior. Other factors, such as HIV serostatus concordance or discrepancy need to be taken into consideration; they will be subject of future analyses.

From a public health perspective, our findings indicate the need to consider the diversity of sexual behaviors, identities, and partners among MSM present in Buenos Aires in surveillance studies and to develop prevention programs tailored to different subpopulations. For example, prevention campaigns acknowledging that some men have sex with women, men, and transvestites may promote more openness in the discussion of the specific prevention needs of different partnerships. Capacity building for health providers to enable them to inquire about such partnerships in a non-judgmental manner may also help to promote prevention.

Lessons derived from this study can be useful to researchers planning on using RDS in the future. First, thorough formative work conducted before launching RDS may still fail to uncover phenomena detected by the survey. This was the case with the surprising finding that one third of our participants reported recent sexual behavior with all three partner types (men, women, and transvestites). In such cases, complementing data obtained through structured surveys with qualitative data obtained through in-depth interviews and field observations may help significantly to understand unexpected outcomes.

Second, the role that incentives play in RDS requires further consideration. The level of monetary incentive that we offered seems to have been insufficient to motivate the participation of members of relatively affluent sectors of the population such as those residing in the North Zone of Buenos Aires. Yet, the incentives may have been too attractive to unemployed and destitute individuals who readily volunteered to participate, at times raising questions about the truthfulness of their answers to the selection criteria. It would be problematic and probably unethical to offer different levels of incentives to people from different socioeconomic or geographic extractions. Different types of incentives of similar value (e.g., entry tickets for gay clubs or meal vouchers) would not solve the problem given that any voucher can still be commercialized (as reported by Johnston, et al. [16]). Maybe incentives should be tailored to the circumstances of less affluent potential participants while the same incentives plus appeals to altruistic motivations are used for more affluent individuals.

Third, Johnston et al. [16] recommend that, to increase the accuracy of the network data collected, questions should include “each component of the eligibility criteria used in the survey.” We believe that this is impractical and probably unfeasible. In our case, participants were not asked to report how many MSM in their network had had sex with a man in the prior six months and at least 10 times in his life, which was part of our eligibility criteria. First, it is unlikely that participants could have such detailed knowledge of most MSM in their network (called “transmission error” by McCormick et al. [27]). Second, this kind of detailed questioning can result in increased masking, i.e., participants becoming clearly aware of the eligibility criteria and consequently alerting possible study candidates on what to say to be enrolled in the study.

Fourth, in a few cases when a referred individual did not qualify, we returned the coupon to the referring participant so that he could give it to another man in his network. This may have resulted in inadvertent disclosure of the eligibility criteria and may have encouraged masking. Yet, the alternative of invalidating the coupon would have cut chains of referrals and may have discouraged further referrals. An option could be to have the screen for eligibility done by CASI without the screening staff being notified of the criteria. This would relieve the staff from the pressure to answer questions about why someone did not qualify (staff can attribute rejections to an algorithm established by the researchers to which the screening staff is blind). Related to this is the issue of coupon expiration (in our study happening after 60 days). Although this time frame may limit the chances of someone participating in the study, the alternative of no expiration date may result in an untenable study duration.

Fifth, RDS removes control from investigators on where and how to recruit study participants and puts it in the hands of participants themselves. This has benefits and drawbacks that should be carefully assessed.

Finally, the pace of development of statistical analysis methods for RDS-collected data has been slower than the explosion of implementation of RDS as a recruitment tool. Issues concerning ways to assess network size, to estimate design effects and their effect on power estimates, and to assess the presence of bottlenecks in the social networks [7] are just a few of the many issues that need further study.

There are some limitations to our data that should be kept in mind. The parameters estimated via RDS samples have greater uncertainty than those from simple random samples as the observations are dependent [28]. Salganik [29] proposed the bootstrap procedure to better account for the uncertainty; however, Goel and Salganik [7] found the procedure to be invalid. To our best knowledge, there is no good way that is currently known to account for the excess variance that results from the RDS sampling design. As stated before, we had relative little representation of residents in the more affluent northern area of Buenos Aires, and this may affect the representativeness of the sample. Furthermore, our study was not designed to explore the sexual identity of participants; therefore, our determination of sexual identity was a crude question that did not explore sexual attraction, group identity, level of community acceptance of sexual diversity, and the like. Yet, we believe, as Coleman [30] states, that “the realities of the world today are that these labels [homosexual, bisexual, and heterosexual] still mean something to people, something very important to their overall self-concept” (p. 19). From that perspective, we consider that the partition of the sample by sexual identity subgroups is reasonable. The trend for those choosing “other” to be younger and less educated may also indicate that the development of sexual identity is related to educational level and years of sexual networking. Within these limitations, our study makes an important contribution to illuminating distinct subgroups within the MSM category that can benefit from further studies.
